# Absorption and Transport of Sea Cucumber Saponins from *Apostichopus japonicus*

**DOI:** 10.3390/md14060114

**Published:** 2016-06-17

**Authors:** Shuai Li, Yuanhong Wang, Tingfu Jiang, Han Wang, Shuang Yang, Zhihua Lv

**Affiliations:** Marine Drug and Food Institute, School of Medicine and Pharmacy, Ocean University of China, Qingdao 266003, China; lishuai890126@126.com (S.L.); yhwang@ouc.edu.cn (Y.W.); jiangtingfu@ouc.edu.cn (T.J.); wanghan0812@sina.com (H.W.); yangshuang@ouc.edu.cn (S.Y.)

**Keywords:** sea cucumber saponins, bioavailability, Caco-2 cells, intestinal perfusion, structure-effect

## Abstract

The present study is focused on the intestinal absorption of sea cucumber saponins. We determined the pharmacokinetic characteristics and bioavailability of Echinoside A and Holotoxin A_1_; the findings indicated that the bioavailability of Holotoxin A_1_ was lower than Echinoside A. We inferred that the differences in chemical structure between compounds was a factor that explained their different characteristics of transport across the intestine. In order to confirm the absorption characteristics of Echinoside A and Holotoxin A_1_, we examined their transport across Caco-2 cell monolayer and effective permeability by single-pass intestinal perfusion. The results of Caco-2 cell model indicate that Echinoside A is transported by passive diffusion, and not influenced by the exocytosis of P-glycoprotein (P-gp, expressed in the apical side of Caco-2 monolayers as the classic inhibitor). The intestinal perfusion also demonstrated well the absorption of Echinoside A and poor absorption of Holotoxin A_1_, which matched up with the result of the Caco-2 cell model. The results demonstrated our conjecture and provides fundamental information on the relationship between the chemical structure of these sea cucumber saponins and their absorption characteristics, and we believe that our findings build a foundation for the further metabolism study of sea cucumber saponins and contribute to the further clinical research of saponins.

## 1. Introduction

Glycosides are an important class of natural products discovered in higher plants and animals [1,2,3]. Sea cucumber saponins are triterpene glycosides isolated from holothuroids (Echinodermata), which consists of a sugar moiety composed of a maximum of six monosaccharides attached to a triterpene based on holostanol [4,5]. With more and more recent papers describing saponins properties, sea cucumber saponins are reported to possess extensive physiological activities [6] including hemolytic, antitumoral, antifungal, anti-bacterial, antiviral, cytostatic, and immunoregulatory activity [7,8,9,10,11,12,13]. However, the pharmacological effect of sea cucumber saponins *in vivo* as an oral medicine depends on its bioavailability in humans. Our attention is focused on two sea cucumber saponins, Echinoside A and Holotoxin A_1_, isolated from *Apostichopus japonicus* which possess wide pharmacological effects, including antifungal, hemolytic, cytotoxic activity, increasing viscidity of cytomembrane, and decreasing lipids in serum [14].

Bioavailability represents the rate and extent of an oral dose reaching the blood circulation. It is controlled by the solubility and dissolution rate of a drug in the intestinal fluid and permeability across the intestinal membrane, pre-systemic metabolism and, sometimes, the efficiency of the drug transporting system [15]. In addition to an animal model, a number of *in vitro* and *in situ* experimental models have been developed to figure out the intestinal permeability of a drug and its mechanism, such as the Caco-2 cell model, single-pass intestinal perfusion, and everted gut sac [16,17,18].

Caco-2 cells originate from human colonic carcinoma. They can spontaneously differentiate into apical side and basolateral side (similarly to the villi and the base of the intestine), and form a monolayer under the culture condition *in vitro* [19,20]. Similarly to the human small intestine, Caco-2 cells express several active transport systemers and marker enzymes [21,22,23]. Single-pass intestinal perfusion is the most frequently used technique, which provides the closest conditions to the *in vivo* oral administration [24,25].

Previously, several studies about the absorption of plant saponins have been published. Jiang *et al.* [26] investigated the bioavailability of Soyasaponin, demonstrated that Soyasaponin I and Sapongenol B have limited absorption. Han *et al.* [27] indicated that transport across Caco-2 cell monolayer for panaxnotoginseng saponin is a simple passive diffusion process and no efflux transporters showed effects on it. Although new sea cucumber saponins with great physiological activity have been discovered successively [28], knowledge on the bioavailability of these compounds is limited.

Our study attempted to characterize the permeability of sea cucumber saponins in the intestine. We investigated the pharmacokinetics of two sea cucumber saponins, and analyzed the relationship between their bioavailability and their chemical structure. Caco-2 monolayer and single-pass intestinal perfusions were used to explore the transport mechanism of sea cucumber saponins.

## 2. Results

### 2.1. Characterization of Echinoside A

**Figure 1:**
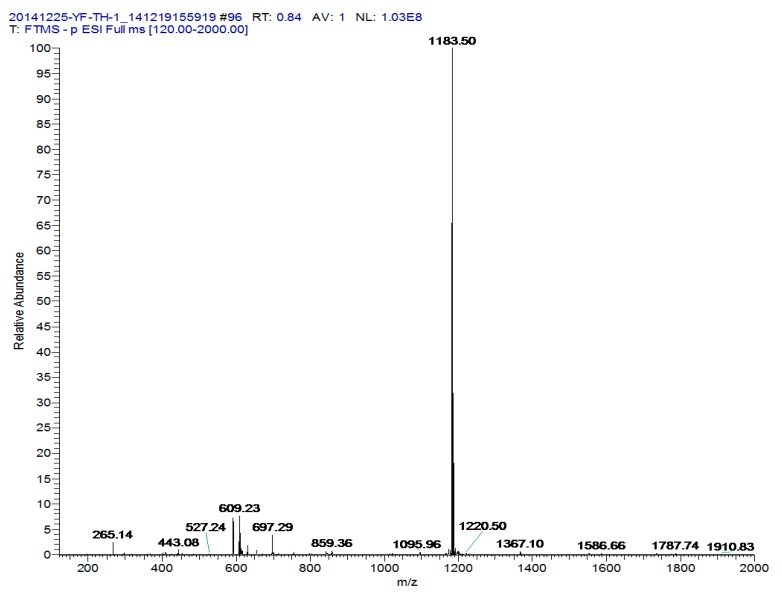
ESI-MS spectra of Echinoside A in negative mode.

Echinoside A was isolated and identified by our research group. Its mass spectrum (MS) and nuclear magnetic resonance (NMR) data are shown in Figure 1 and Table 1 and Table 2. This is the first characterization of this saponin in *Apostichopus japonicas*.

**Figure 2:**
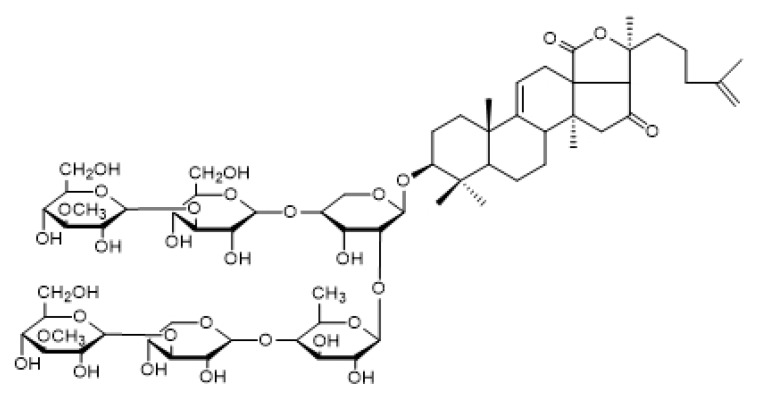
Chemical structure of Holotoxin A_1_.

**Figure 3:**
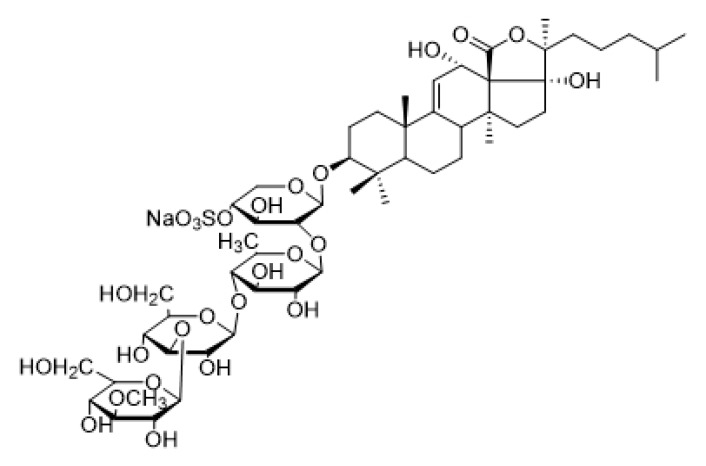
Chemical structure of Echinoside A.

The chemical structure of Holotoxin A_1_ and Echinoside A are shown in Figure 2 and Figure 3.

### 2.2. Method Validation

**Figure 4:**
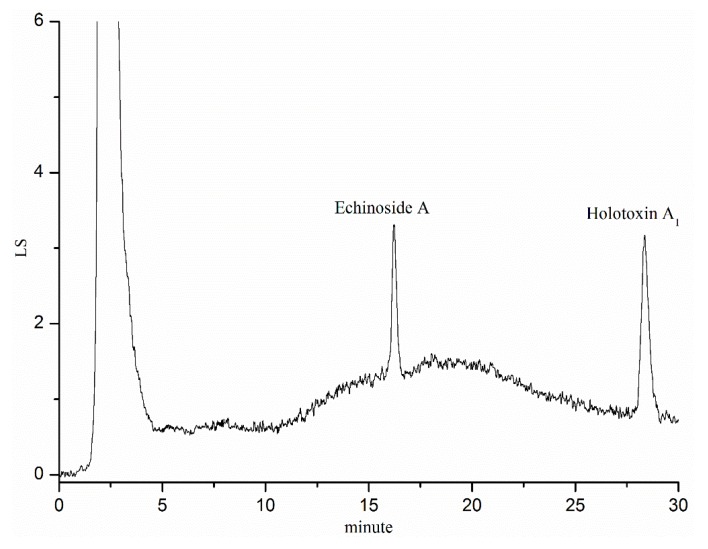
HPLC-chromatogram of Echinoside A and Holotoxin A_1_.

The retention times for Echinoside A and Holotoxin A_1_ were 16.2 and 28.3 min, respectively (Figure 4).

#### 2.2.1. Linearity and Sensitivity

The calibration curves were linear over the concentration range of 100–5000 ng/mL and 100–20,000 ng/mL for Echinoside A and Holotoxin A_1_ in Hank’s balanced salt solution (HBSS) and rat plasma. The regression equation for the calibration curve of Echinoside A in HBSS and plasma were, respectively, *Y* = 528884*X* − 2318.7 (*r*^2^ = 0.9988) and *Y* = 30668*X* + 30.707 (*r*^2^ = 0.9991). The regression equation for the calibration curve of Holotoxin A_1_ in HBSS and plasma, were respectively, *Y* = 25428*X* − 301.05 (*r*^2^ = 0.9991) and *Y* = 25562*X* + 2171.8 (*r*^2^ = 0.9992). The lower limits of quantification (LLOQ) of Echinoside A and Holotoxin A_1_ in HBSS and rat plasma were found to be 100 ng/mL. The limit of detection (LOD) for Echinoside A and Holotoxin A_1_ in HBSS and plasma were both 50 ng/mL. Meanwhile, the calibration curves were linear over the concentration range of 500–15,000 ng/mL for Echinoside A and Holotoxin A_1_ in perfusate. The regression equation for the calibration curve of Echinoside A and Holotoxin A_1_ in perfusate were, respectively, *Y* = 39950*X* + 576.33 (*r*^2^ = 0.9990) and *Y* = 46916*X* + 1715.7 (*r*^2^ = 0.9989). The LLOQ of Echinoside A and Holotoxin A_1_ were found to be 500 ng/mL in perfusate. The LOD for Echinoside A and Holotoxin A_1_ in perfusate were 200 ng/mL.

#### 2.2.2. Precision and Accuracy

The precision and accuracy data are shown in Table 3. The repeatability of the method was examined by both intra and inter-day repeatability. The intra and inter-day R.S.D.% of Echinoside A and Holotoxin A_1_ were less than 7.8% and 5.6%, and accuracy was ranged from −4.4% to 5.8%. The results indicated the stability and repeatability of the method for the quantitative determination of Echinoside A and Holotoxin A_1_.

#### 2.2.3. Recovery and Stability

As shown in Table 4, the absolute recovery of Echinoside A and Holotoxin A_1_ in plasma were both above 90%. Two analytes were stable after frozen storage, analysis processing, and freeze-thaw conditions. The stability of Echinoside A and Holotoxin A_1_ in plasma and perfusate were 93.4%–102.4% and 88.2%–94.8% under three conditions, respectively (Table 5), which confirms the stability of Echinoside A and Holotoxin A_1_.

### 2.3. Pharmacokinetic Analysis of Sea Cucumber Saponins

**Figure 5:**
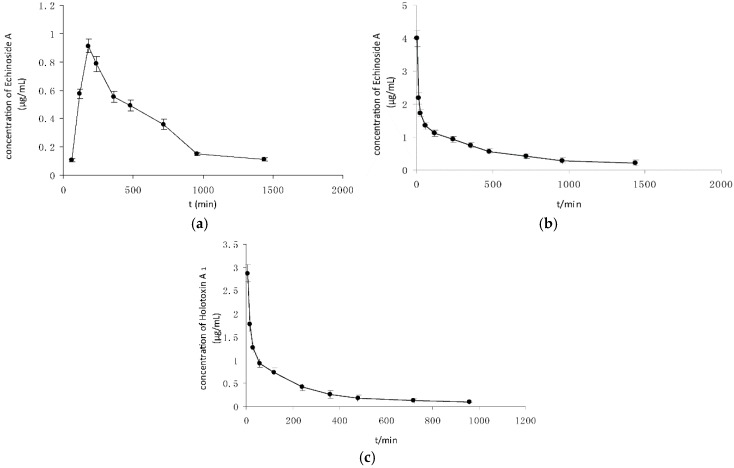
(**a**) Plasma concentrations-time curves of Echinoside A after oral administration; (**b**) plasma concentrations-time curves of Echinoside A after intravenous administration; and (**c**) plasma concentrations-time curves of Holotoxin A_1_ after intravenous administration. (means ± SD, *n* = 5).

After oral administration, Echinoside A and Holotoxin A_1_ were present in blood with different levels. Figure 5a shows the mean plasma concentrations-time profiles of Echinoside A. After oral administration, Echinoside A was detected at 1 h in rat plasma, which indicated that it could be gradually absorbed into plasma. Within 3 h, the concentration of Echinoside A in the plasma achieved the max value of 910 ng/mL. The elimination half-life time (*T*_1/2_) of Echinoside A was 6.99 h. On the contrary, no saponin was detected during the experiment (*i.e.*, 24 h). The relevant pharmacokinetic parameters are listed in Table 6.

After intravenous administration, Echinoside A and Holotoxin A_1_ were present in plasma and decreased gradually over time (Figure 5b,c). The concentration-time curve of Echinoside A after intravenous administration included decreasing process only, whereas the concentration-time curve of Echinoside A after oral administration divided into increasing and decreasing trend. The relevant pharmacokinetic parameters are listed in Table 6. The area under the curve (AUC) and *C*_max_ of Echinoside A after intravenous administration were significantly higher (*p* < 0.05) than Holotoxin A_1_.

Echinoside A and Holotoxin A_1_ exhibited different transport dynamics after oral and intravenous administration. The bioavailability of Echinoside A was 59%, meanwhile Holotoxin A_1_ was not detected in the plasma after oral administration. This finding revealed that the bioavailability of Holotoxin A_1_ was lower than Echinoside A.

### 2.4. Transport Experiments of the Caco-2 Cell Model

**Figure 6:**
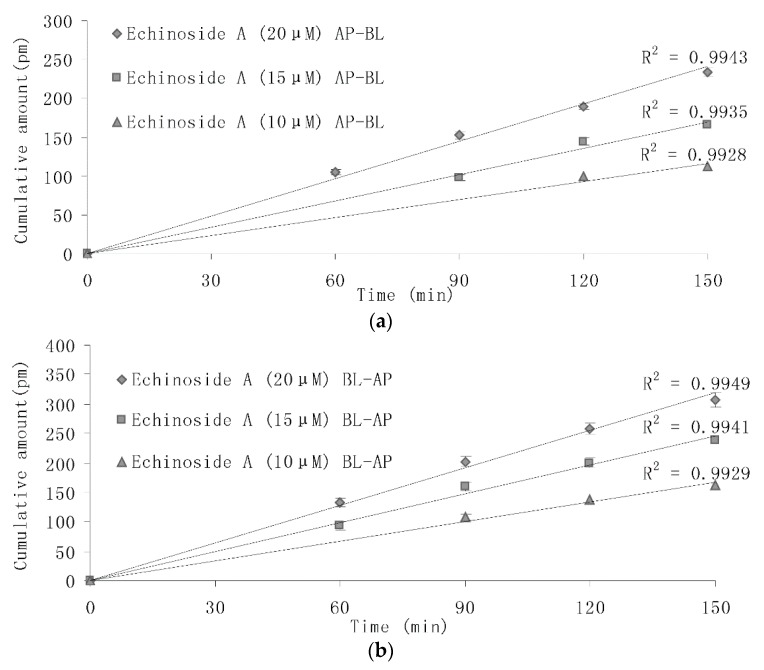
(**a**) AP-to-BL transport of different concentration of Echinoside A across Caco-2 monolayers; and (**b**) BL-to-AP transport of different concentration of Echinoside A across Caco-2 monolayers, (means ± SD, *n* = 3).

The present study was undertaken to detect the transport of sea cucumber saponins with different concentrations over time. Figure 6 shows Echinoside A collected in the receiver chamber at 0, 60, and 150 min. The accumulation of different concentration of Echinoside A transported from apical-to-basolateral (AP-to-BL) (Figure 6a) and from basolateral-to-apical (BL-to-AP) (Figure 6b) increased linearly within 150 min. The results indicated that the cumulative amounts in the receiver chamber rose significantly (*p* < 0.05) at the same time as the concentration of Echinoside A increased; meanwhile, the cumulative amounts in the receiver chamber in BL-AP direction were significantly higher (*p* < 0.05) than the cumulative amounts in AP-BL direction at the same time in each concentration.

**Figure 7:**
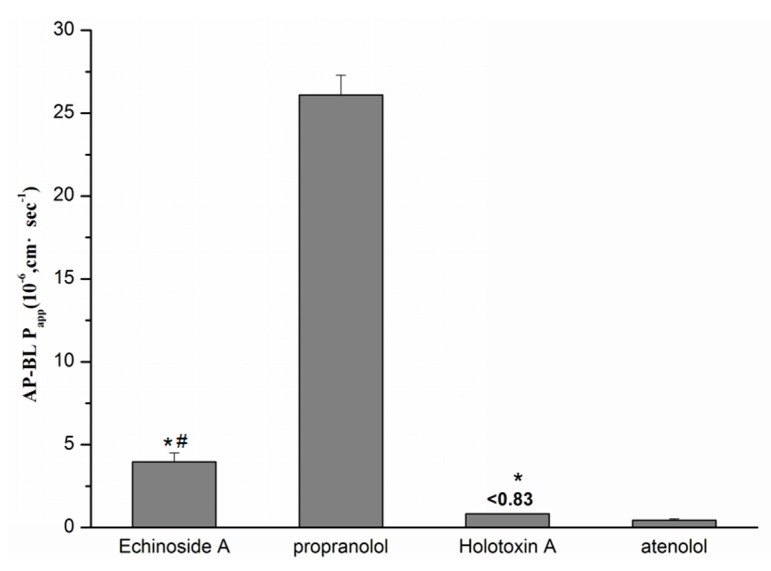
Papp (AP to BL) value of Echinoside A, Holotoxin A_1_ and two transcellular transport markers, propranolol and atenolol, (means ± SD, *n* = 5). * *p* < 0.05, compared with propranolol; # *p* < 0.05, compared with atenolol.

The absorption and transport of the drug in Caco-2 cell monolayer was evaluated by the apparent permeability coefficients (Papp). The PappAB (Papp (AP to BL) value) of Echinoside A (20 µM), Holotoxin A_1_ (20 µM) and two transcellular transport markers propranolol and atenolol are summarized in Figure 7. Figure 7 indicated that the PappAB of Echinoside A was significantly higher (*p* < 0.05) than atenolol, and significantly lower (*p* < 0.05) than propranolol. The PappAB of Holotoxin A_1_ was significantly lower (*p* < 0.05) than propranolol and Echinoside A.

The PappAB of Echinoside A was (3.96 ± 0.55) × 10^−6^ cm·s^−1^, which was about one sixth of that for propranolol, at (26.1 ± 1.2) × 10^−6^ cm·s^−1^; meanwhile, it was more than eight-fold higher than that for atenolol of (0.48 ± 0.07) × 10^−6^ cm·s^−1^. Holotoxin A_1_ was not detected in the receiver compartment, which indicated that Holotoxin A_1_ accumulated in the receiver compartment was less than the limit of detection; therefore, the PappAB of Holotoxin A_1_ was less than 0.83 × 10^−6^ cm·s^−1^.

At the same time, Echinoside A and Holotoxin A_1_ in the AP chambers and cells were detected to verify their stability. The percentage recovered in AP chambers, BL chambers, and cell monolayer are shown in Table 7. Table 7 indicated that Echinoside A were present in AP chambers, BL chambers, and cell monolayer. Holotoxin A_1_ was present in AP chambers. The recovery revealed that they were stable in this model.

P-glycoprotein (P-gp) is expressed in the apical side of Caco-2 monolayers with verapamil as the classic inhibitor. The efflux ratio which is used to present the extent of excretion is calculated by efflux *versus* absorption (PappBA/PappAB). To investigate the present of exocytosis, transport experiment was examined in the presence and absence of verapamil (Sigma-Aldrich, St. Louis, MO, USA, 100 μM). Table 8 showed the Papp value of Echinoside A (20 µM) and Holotoxin A_1_ (20 µM) in the absence and presence of verapamil. The lack of directional preference and no significant decrease of the efflux ratio compared to the data in the absence of the P-gp-inhibitor suggested that Echinoside A was transported by passive diffusion without the participation of P-gp.

We found that the Caco-2 cell model showed high and efficient transport of Echinoside A, and oppositely, poor transport of Holotoxin A_1_. The results were coincident with their transport dynamics parameters of pharmacokinetics.

### 2.5. Single-Pass Intestinal Perfusion of Sea Cucumber Saponins

**Figure 8:**
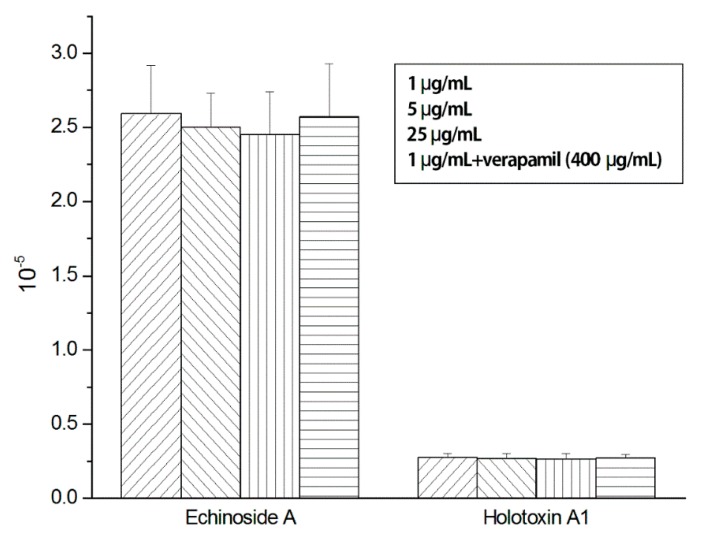
Peff values of Echinoside A and Holotoxin A_1_ at 1, 5, 25 μg/mL, as well as 50 μg/mL with the addition of verapamil using single-pass intestinal perfusion (means ± SD, *n* = 5).

After the perfusion of different doses of Echinoside A and Holotoxin A_1_, their steady-state concentrations collected from the outlet were detected. The intestinal permeability of Echinoside A and Holotoxin A_1_ were determined by permeability across the intestinal membrane (Peff) value. The Peff of Echinoside A and Holotoxin A_1_ from *in situ* vascular perfusion were, respectively, 2.50 × 10^−5^ cm·s^−1^ and 2.67 × 10^−6^ cm∙s^−1^. The Peff values of each concentration are listed in Figure 8.

Concentration-dependent changes of Echinoside A and Holotoxin A_1_ in permeability were not found in the segment of intestine. The permeability at 25 μg/mL of Echinoside A was close to that at 1 μg/mL. No significant increase in permeability of Echinoside A and Holotoxin A_1_ was tested when verapamil (400 μg/mL) was added to the perfusate, which indicated that P-gp did not affect the intestinal absorption of Echinoside A and Holotoxin A_1_.

The results of single-pass intestinal perfusion model on the absorption and metabolism of Echinoside A and Holotoxin A_1_ confirmed the results of pharmacokinetics experiments and transport experiments across Caco-2 model.

### 2.6. Formatting of Mathematical Components

Papp = (d*Q/*d*t*)/(*A* × *C*_0_) (1)

where, d*Q/*d*t* (nmol·s^−1^) represents the rate of the compound accumulated in the receiver compartment over time. *A* (cm^2^) represents the membrane area of the insert. *C*_0_ (nmol/cm^3^) represents the initial concentration of the drug in the donor compartment [29,30,31]. Data were expressed as the means ± SD of three determinations:

Peff = −*Q* ln [*C*_out_*/C*_in_]/2π*rl* (2)

In which *C*_in_ is the inlet concentration and *C*_out_ is the outlet concentration of compound, which is corrected for volume change in the segment using phenol red concentration in the inlet and outlet tubing. *Q* is the flow rate (0.2 mL/min), *r* is the rat intestinal radius (0.18 cm), and *l* is the length of the segment [32].

## 3. Discussion

The current study developed a highly-sensitive and effective HPLC method with evaporative light scattering detection to determine sea cucumber saponins. This method was validated to confirm high accuracy, precision, and reproducibility. This study highlighted the absorption and transport of two sea cucumber saponin monomers using the Caco-2 monolayer model, single-pass intestinal perfusion model, and *in vivo* pharmacokinetics in rats, and speculated the relationship between the chemical structure of two sea cucumber saponins and their bioavailability.

Echinoside A showed higher bioavailability than Holotoxin A_1_. Factors affect the bioavailability of drugs include structural and chemical properties of molecules, such as molecular weight, hydrogen performance, branched chain, and solubility. As a small molecular substance, sea cucumber saponins are neutral, liposoluble, but not hydrophobic. Compared with Holotoxin A_1_, Echinoside A has a lower molecular weight. Holotoxin A_1_ has six monosaccharides, which is two more than that of Echinoside A. Additionally, the six monosaccharides are divided into two branched chains, which results in larger steric hindrance. We speculate that these reasons lead to lower bioavailability of Holotoxin A_1_.

Artursson, Karlsson [33], and Lau [34] indicated that drugs with Papp ≥ 3 × 10^−6^ cm·s^−1^ represented high permeability and good absorption, while compounds with a Papp of less than about 2 × 10^−6^ cm·s^−1^ exhibited poor oral absorption. The results of the Caco-2 cell model and single-pass intestinal perfusion model showed high and efficient transport of Echinoside A, and oppositely, poor transport of Holotoxin A_1_, which confirmed the results of pharmacokinetics experiments, and demonstrated our conjecture that the differences in chemical structure between compounds was a factor that explained their different characteristics of transport across the intestine.

Jiang *et al.* [27] investigated the bioavailability of Soyasaponin, demonstrated that Soyasaponin I and Sapongenol B have limited absorption. Han *et al.* [27] used Caco-2 cells and rat models to study the mechanism of absorption after oral administration of panaxnotoginseng saponins (PNS). The results indicated that PNS showed low membrane permeability on Caco-2 cell monolayer and poor absorption. Our study indicated that Echinoside A exerted efficient transport on the Caco-2 cell monolayer and favorable bioavailability compared with plant saponins.

Processed sea cucumbers (trepangs) have a high commercial value and are consumed for food and traditional medicine in Asian communities. Caulier *et al.* [35] compared the saponins contained in the body wall of sea cucumbers and the trepangs. They found that saponins seem to be thermically resistant in the processed procedure which indicated that sea cucumbers saponins were stable as food.

Our work found a sea cucumber saponin monomer with high and efficient transport, which indicated that this secondary metabolite with extensive physiological activities can be absorbed efficiently by humans. This study lays a foundation for further study of the clinical application of sea cucumber saponins. In addition, our speculation about the relationship between the chemical structure of two sea cucumber saponins and their bioavailability could be verified by more sea cucumber saponins in the future studies and provided a method for direct assessment of the intestinal absorption of drug.

## 4. Materials and Methods

### 4.1. Chemicals and Materials

The human colon adenocarcinoma cell line, Caco-2, was obtained from the cell resource center of the Shanghai Institutes for Biological Sciences. Dulbecco’s modified eagle medium (DMEM) and fetal bovine serum were from Gibco (Thermo Fisher, Shanghai, China), penicillin and streptomycin were from Sigma-Aldrich.

Sea cucumber saponins were separated and purified by our research group [36]. We used two saponin monomers (Holotoxin A_1_ and Echinoside A) which did not contain other molecules in our experiments. 15.5 kg sea cucumbers were used for extraction. The weight of extraction was 9.763 g. The productive rate of Holotoxin A_1_ and Echinoside A from the extraction were respectively about 6% and 4%.

Millicell hanging cell culture inserts (polyethylene terephthalate membrane, 0.33 cm^2^ surface, 0.4 μm pore size) were purchased from Corning (New York, NY, USA). All other chemicals used in this study were of analytical grade.

Hanks’ buffered saline solution (HBSS) containing 10 mM HEPES and 25 mM D-(+)-glucose. Perfusate was Krebs-Ringer buffer solution containing (g/L) NaCl 7.8, KCl 0.35, CaCl_2_ 0.37, MgCl_2_ 0.22, NaH_2_PO_4_ 0.32, glucose 3.0, NaHCO_3_ 1.37, dextran 30, and BSA 50.

### 4.2. Animals

Male Wistar rats weighting 200–220 g were obtained from Qingdao Institute for Drug Control (Qingdao, China, SCXK2009007) and acclimated for 7 d in an environmentally-controlled room (temperature: 25 ± 2 °C, humidity: 50% ± 5%, 12 h dark-light cycle) with free access to water and food. All *in vivo* animal work was approved by Animal Ethics Committee of School of Medicine and Pharmacy, Ocean University of China. The rats were subjected to fasting, with access to water, for 12 h before the experiment in order to avoid the influence of the food to the absorption of sea cucumber saponins. Five rat individuals were used for each parallel control group.

### 4.3. Chromatographic Conditions

The content of sea cucumber saponins was tested by HPLC system (Waters Corp, Milford, MA, USA) consisting of a pump (Model 1525), an autosampler (Model 717), and an evaporative light-scattering detector (ELSD, Model 2420). Separations were performed on a Kromasil C18 (Zonran Technologies, Huaian, China) reversed-phase column (5 μm, 150 × 4.6 mm). The column temperature was set to 40 °C and the flow rate was 0.7 mL·min^−1^. The mobile phase consisted of double-distilled water (solution A) and acetonitrile (solution B), the acetonitrile gradient was 27% from 0 to 15 min, 27%–42% from 15 to 30 min, 42%–27% from 30 to 32 min. The carrier gas pressure of ELSD was 25 psi, the drift tube temperature was 60 °C, the atomization efficiency was 50% and the gain value was 25. These conditions were chosen according to the research of Zhang *et al.* [36].

### 4.4. Method Validation

Stock solutions of Echinoside A and Holotoxin A_1_ were prepared in water (1 mg/mL) and working standard solutions were prepared by serial dilution in HBSS, blank rat plasma, and blank perfusate, respectively. The plasma with the quality control (QC) sample was blended with methanol and centrifuged for 10 min at 15,000 rpm, the supernatant was dried under nitrogen and dissolved with methanol for analysis.

#### 4.4.1. Linearity

The linearity of HPLC method for the determination of Holotoxin A_1_ and Echinoside A was evaluated by calibration curves. The calibration curves were obtained by plotting the chromatographic peaks area *versus* the concentrations of analytes prepared. The slope, intercept, and correlation coefficient of the calibration curves were determined by least squares linear regression analysis.

#### 4.4.2. Precision and Accuracy

Five QC samples of different concentrations were injected on a single day (intra-day) and on three consecutive days (inter-day). The precision was expressed as the relative standard deviation (R.S.D.%) and the accuracy was expressed as the relative error (R.E.%).

#### 4.4.3. Recovery and Stability

Five replicates of Holotoxin A_1_ and Echinoside A diluted to 100, 1000, and 5000 ng/mL by plasma and perfusate were detected, and the absolute recovery was determined by comparing the peak-area value from the calibration curve and the real concentration. Freeze-thaws, short-term, and long-term stabilities of Holotoxin A_1_ and Echinoside A in plasma and perfusate were verified using different concentrations of QC samples. In the freeze-thaw cycle, the samples were frozen and stored at −20 °C for 24 h, then thawed at ambient temperature. To evaluate long-term stability, samples were kept at ambient temperature for 5 d until extraction. For the short-term stability, samples were kept at ambient temperature for 24 h before extraction.

### 4.5. In Vivo Pharmacokinetics of Sea Cucumber Saponins in Rats

#### 4.5.1. Oral Administration of Sea Cucumber Saponins

20 mg/kg Echinoside A and Holotoxin A_1_ dissolved in water were, respectively, orally administered to the rats by gavage. Blood samples were collected from the orbital cavity at different times (5, 15, 30 min and 1, 2, 3, 4, 8, 12, and 24 h). The plasma was obtained from the blood samples by being centrifuged for 10 min at 4000 rpm, and then frozen at −20 °C prepared for analysis.

#### 4.5.2. Intravenous Administration of Sea Cucumber Saponins

20 mg/kg Echinoside A and Holotoxin A_1_ dissolved in water were, respectively, orally administered to the rats by tail vein injection. Blood samples were collected from the orbital cavity at different times (5, 15, 30 min and 1, 2, 4, 8, 12, and 24 h). The plasma was separated and frozen at −20 °C and prepared for analysis.

We chose these parameters after we referred to other saponins. Doses of administration for saponins are different in previous research. Li *et al.* [37] investigated the pharmacokinetics of ilexsaponin A_1_ in rats, the dose of intravenous administration was 30 mg/kg. Odani *et al.* [38] investigated the pharmacokinetics of ginsenoside Rg_1_ in rats, the dose of intravenous administration was 5 mg/kg, and the dose of oral administration was 100 mg/kg. Lin *et al.* [39] investigated the pharmacokinetics of ginsenoside in rats, with the dose of oral administration being 50 mg/kg.

### 4.6. Caco-2 Cells Model

#### 4.6.1. Cell Culture

Caco-2 cells were cultured in DMEM supplemented with 20% fetal bovine serum, 100 U/mL penicillin, and 100 μg/mL streptomycin. Cells were grown in a humidified atmosphere of 5% CO_2_ at 37 °C and were subcultured at 80%–90% confluency.

#### 4.6.2. Transepithelial Transport Experiments

For transport studies, Caco-2 cells were seeded in Millicell (Corning) hanging cell culture inserts (0.33 cm^2^ surface, 0.4 μm pore size) at a density of 1 × 10^5^/cm^2^. The basolateral and apical compartments contained 1.0 and 0.5 mL of culture medium, respectively. Culture medium was replaced three times a week for 14 d, and daily thereafter. Cells with passage numbers 30–65 were used for transport experiments 19–30 d post-seeding. Monolayers with TEER value equal or above 300 Ω·cm^−2^ were selected for transport experiments.

The monolayers were washed twice with preheating transport medium (HBSS) and prei-ncubated for 30 min. Transport medium containing different concentration of sea cucumber saponins was added on either the apical (0.5 mL) or basolateral (1 mL) side while the receiver chamber contained corresponding volume of transport medium. Samples were collected from the receiver chamber after 30, 60, 90, 120, and 150 min. During the experiments, each sampling volume (300 μL) was replaced by an equal volume of blank transport medium. The relevant parameters were chosen according to previous research [30,40,41].

Samples collected from the receiver chamber were detected and the apparent permeability coefficients (Papp) were calculated. The absorption and transport of the drug in Caco-2 cell monolayer was evaluated by Papp. Propranolol and atenolol are typical drugs with high and low permeability on the Caco-2 cell monolayer. The Papp of propranolol and atenolol measured in this study were, respectively, (26.1 ± 1.2) × 10^−6^ and (0.48 ± 0.07) × 10^−6^ cm∙s^−1^, which was coincident with the literature [42,43].

### 4.7. Single-Pass Intestinal Perfusion Model

The rats were anaesthetized with an intra-abdominal injection of 10% chloral hydrate at a dose of 5 mL/kg. The enterocoelia of the rat was dissected by a 3–4 cm midline length, a segment of intestine about 10 cm long was separated and washed with saline. Silicone tubing was inserted into both sides of the segment of intestine and a peristaltic pump was connected to the proximal side of the tube. A pledget with saline was added onto the surgical area to avoid loss of fluid. The segment was cleared with 37 °C saline firstly, then blank K-R buffer solution was infused at the inlet and blank perfusate was collected at the outlet. The segment was infused with perfusate containing phenol red and different concentrations of sea cucumber saponins (1, 5, and 25 μg/mL) at a flow rate of 2 mL/min. Then the rate was reduced to 0.2 mL/min for 30 min until steady-state. Samples were obtained from the outlet of the segment every 15 min and stored at −20 °C for analysis. Different concentrations of sea cucumber saponins were perfused to highlight the absorption mechanism of sea cucumber saponins, which were investigated by the concentration-dependent change of the permeability values. 400 μg/mL verapamil was added to the perfusion solution containing 5 μg/mL sea cucumber saponins and 20 μg/mL phenol red to investigate whether the intestinal absorption of sea cucumber saponins were affected by P-gp. The relevant parameters were chosen according to previous researches [18,44].

### 4.8. Statistical Analysis

Each experiment was carried out more than three individual times. The differences of AUC and *C*_max_ between Echinoside A and Holotoxin A_1_ after intravenous administration were assessed by a one-way analysis of variance (ANOVA) test using the SPSS (version 18.0, IBM SPSS Inc., Chicago, IL, USA). In the absorption transport experiment, two factor ANOVA for concentration and direction were used to compare the cumulative amounts in bidirectional transport. A pair-wise test was used to compare the Papp values of Echinoside A, Holotoxin A_1_, propranolol and atenolol. Differences were considered statistically significant at *p* < 0.05.

## 5. Conclusions

In this study, a highly-sensitive and effective HPLC method with evaporative light scattering detection was developed to determine sea cucumber saponins. This method was validated to confirm high accuracy, precision, and reproducibility. The method was successfully applied to the analysis in Caco-2 monolayer model, single-pass intestinal perfusion model, and *in vivo* pharmacokinetics in rats. The result indicated that Echinoside A showed higher bioavailability in rats than Holotoxin A_1_. In the Caco-2 monolayer model, the Papp value demonstrated efficient transport of Echinoside A and poor transport of Holotoxin A_1_, and the single-pass intestinal perfusion model confirmed high permeability of Echinoside A and poor permeability of Holotoxin A_1_. This study highlighted the absorption and transport of two sea cucumber saponin monomers, the relationship between the chemical structure of sea cucumber saponins and their bioavailability and the clinical application of sea cucumber saponins will be studied further in the future.

## Figures and Tables

**Table 1:** ^1^H NMR and ^13^C NMR data for the aglycone moiety of Echinoside A.

Position	δ_C_	δ_H_ (*J* in Hz)	Position	δ_C_	δ_H_ (*J* in Hz)
1	36.9 t	1.54 m	15	37.2 t	1.89 m
		1.88 m			1.42 m
2	27.6 t	1.90 m	16	36.4 t	2.37 d (11.7)
		2.14 m			2.70 m
3	89.2 d	3.39 d (10.9)	17	89.8 s	-
4	40.5s	-	18	175.3 s	-
5	53.2 d	1.10 s	19	23.5 q	1.28 s
6	21.7 t	1.36 m	20	87.7 s	-
		1.54 m	21	23.6 q	1.76 s
7	28.8 t	1.54 m	22	39.5 t	1.85 m
		1.76 m	23	22.8 t	1.80 m
8	41.4 d	3.39 d (10.9)	24	40.2 t	1.18 m
9	154.5 s	-	25	28.5 d	1.54 m
10	40.2 s	-	26	23.2 q	0.86 s
11	116.1 d	5.63 d (4.2)	27	23.3 q	0.87 m
12	71.8 d	5.05 m	30	28.5 q	1.28 s
13	59.1 s	-	31	17.2 q	1.13 s
14	46.9 s	-	32	20.6 q	1.70 s

**Table 2:** ^1^H NMR and ^13^C NMR data for the sugar moiety of Echinoside A.

Position	δ_C_	δ_H_ (*J* in Hz)	Position	δ_C_	δ_H_ (*J* in Hz)
Xyl	-	-	Glc	-	-
1	105.8	4.71	1	105.5	4.96
2	83.8	4.02	2	72.2	4.02
3	76.2	4.26	3	88.4	3.88
4	74.4	5.12	4	65.3	4.23
5	62.8 t	3.67	5	76.9	4.26
		4.40	6	61.3 t	4.30
-	-	-			4.55
Qui	-	-	MeGlc	-	-
1	105.9	5.05	1	106.2	5.32
2	74.5	4.02	2	76.3	4.02
3	75.6	4.17	3	89.8	4.23
4	87.9	3.75	4	70.4	4.19
5	71.9	3.75	5	78.5	3.88
6	18.7	1.72	6	62.7	4.26
-	-	-	OMe	61.3	3.86

**Table 3:** Intra- and inter-day precision and accuracy of Echinoside A and Holotoxin A_1_ measurements in rat plasma (*n* = 5).

Compounds	Nominal Conc. (ng/mL)	Intra-Day			Inter-Day		
		Measured Conc. (mean ± S.D., ng/mL)	Accuracy (R.E.%)	Precision (R.S.D.%)	Measured Conc. (mean ± S.D., ng/mL)	Accuracy (R.E.%)	Precision (R.S.D.%)
Echinoside A	100.00	95.60 ± 4.90	−4.4	5.1	96.20 ± 5.08	−3.2	5.3
	1000.00	985.92 ± 27.51	−1.4	2.8	1023.65 ± 40.33	2.4	3.9
	5000.00	4931.22 ± 94.00	−1.4	1.9	5112.43 ± 71.88	2.2	1.4
Holotoxin A_1_	100.00	104.89 ± 7.60	4.9	7.2	105.75 ± 4.09	5.8	3.9
	1000.00	1029.80 ± 39.89	3.0	3.9	1030.41 ± 31.51	3.0	3.6
	5000.00	4902.63 ± 114.78	−2.0	2.3	4826.33 ± 87.15	−3.5	1.8

**Table 4:** Absolute recovery of the method for determining the concentration of Echinoside A and Holotoxin A_1_ in plasma samples (*n* = 5).

Compounds	Concentration (ng/mL)	Absolute Recovery (mean ± SD %)	CV%
Echinoside A	100	91.30 ± 5.92	8.70
	1000	94.74 ± 5.51	5.26
	5000	95.66 ± 4.08	4.34
Holotoxin A_1_	100	90.58 ± 6.72	9.42
	1000	96.13 ± 4.55	3.87
	5000	95.96 ± 4.80	4.04

**Table 5:** Stability of Echinoside A and Holotoxin A_1_ in rat plasma and perfusate (*n* = 5).

Compounds	Nominal Conc. (ng/mL)	Accuracy (%)					
		Freeze-Thaw Stability		Short-Term Stability		Long-Term Stability	
		Perfusate	Plasma	Perfusate	Plasma	Perfusate	Plasma
Echinoside A	100.00	91.6	94.4	90.8	95.4	89.2	97.2
	1000.00	91.9	96.7	91.3	96.0	92.6	102.1
	5000.00	93.5	98.9	94.4	102.4	91.1	100.3
Holotoxin A_1_	100.00	88.2	93.4	92.7	96.2	92.4	94.7
	1000.00	92.1	101.4	90.9	99.3	91.3	98.6
	5000.00	94.3	98.0	93.8	96.5	94.8	99.6

**Table 6:** Pharmacokinetic parameters of Echinoside A and Holotoxin A_1_ after oral administration and intravenous administration (means ± SD, *n* = 5).

Compounds	Administration	Pharmacokinetic Parameters			
		*T*_1/2_ (h)	*T*_max_ (h)	*C*_max_ (µg/mL)	AUC (h·μg/mL)
Echinoside A	oral	6.9 ± 0.32	3.0	0.91 ± 0.02	9.27 ± 0.39
	intravenous	8.52 ± 0.32	0.08	4.0 ± 0.44 *	16.43 ± 0.45 *
Holotoxin A_1_	intravenous	4.4 ± 0.06	0.08	2.87 ± 0.17	6.53 ± 0.15

* *p* < 0.05, compared with Holotoxin A_1_.

**Table 7:** Percentage of Echinoside A and Holotoxin A_1_ recovered in AP chambers, BL chambers, and cell monolayer (means ± SD, *n* = 5).

Time (min)	Echinoside A			Recovery	Holotoxin A_1_		
	AP Chamber (%)	BL Chamber (%)	Cell Fraction (%)		AP Chamber (%)	BL Chamber (%)	Cell Fraction (%)
60	82.14 ± 2.85	1.04 ± 0.12	14.22 ± 1.14	97.40 ± 2.76	97.66 ± 2.02	-	-
90	81.63 ± 2.04	1.51 ± 0.11	15.05 ± 1.29	98.19 ± 1.97	96.56 ± 1.89	-	-
120	80.25 ± 2.67	1.90 ± 0.20	14.43 ± 1.24	96.58 ± 2.33	95.46 ± 1.72	-	-
150	79.38 ± 2.21	2.36 ± 0.21	14.78 ± 0.92	96.52 ± 2.04	95.90 ± 2.27	<0.2	<0.2

**Table 8:** Values of apparent permeability coefficients (Papp) and efflux ratios in the absence and presence of verapamil (means ± SD, *n* = 5).

Compounds	Papp (× 10^−6^, cm·s^−1^)		Efflux Ratio
	AP-BL	BL-AP	
Echinoside A (20 μM)	3.96 ± 0.55	5.38 ± 0.81	1.36 ± 0.17
Echinoside A (20 μM) + verapamil	4.04 ± 0.39	5.33 ± 0.70	1.32 ± 0.16
Holotoxin A_1_ (18 μM)	<0.83	<0.83	-
Holotoxin A_1_ (18 μM) + verapamil	<0.83	<0.83	-
